# Using real-world data to dynamically predict flares during tapering of biological DMARDs in rheumatoid arthritis: development, validation, and potential impact of prediction-aided decisions

**DOI:** 10.1186/s13075-022-02751-8

**Published:** 2022-03-23

**Authors:** Matthijs S. van der Leeuw, Marianne A. Messelink, Janneke Tekstra, Ojay Medina, Jaap M. van Laar, Saskia Haitjema, Floris Lafeber, Josien J. Veris-van Dieren, Marlies C. van der Goes, Alfons A. den Broeder, Paco M. J. Welsing

**Affiliations:** 1 Department of Rheumatology & Clinical Immunology, University Medical Center Utrecht, Utrecht University, Heidelberglaan 100, 3584 CX Utrecht, The Netherlands; 2 Department of Digital Health, University Medical Center Utrecht, Utrecht University, Heidelberglaan 100, 3584 CX Utrecht, The Netherlands; 3 Central Diagnostic Laboratory, University Medical Center Utrecht, Utrecht University, Heidelberglaan 100, 3584 CX Utrecht, The Netherlands; 4 Reumazorg Zuid-West Nederland, Van Hertumweg 17 – 19, 4462 EV Goes, The Netherlands; 5 Department of Rheumatology, Meander Medical Center, Maatweg 3, 3813 TZ Amersfoort, The Netherlands; 6 Department of Rheumatology, Sint Maartenskliniek, Hengstdal 3, 6574 NA Ubbergen, The Netherlands

**Keywords:** Rheumatoid arthritis, Predictive algorithm, Tapering bDMARD therapy, Applied data analytics in medicine, Biologicals

## Abstract

**Background:**

Biological disease-modifying antirheumatic drugs (bDMARDs) are effective in the treatment of rheumatoid arthritis. However, as bDMARDs may also lead to adverse events and are expensive, tapering them is of great clinical interest. Tapering according to disease activity-guided dose optimization (DGDO) does not seem to affect long term remission rates, but flares are frequent during this process. Our objective was to develop a model for the prediction of flares during bDMARD tapering using data from routine care and to evaluate its potential clinical impact.

**Methods:**

We used a joint latent class model to repeatedly predict the probability of a flare occurring within the next 3 months. The model was developed using longitudinal data on disease activity (DAS28) and other routine care data from two clinics. Predictive accuracy was assessed in cross-validation and external validation was performed with data from the DRESS (Dose REduction Strategy of Subcutaneous tumor necrosis factor inhibitors) trial. Additionally, we simulated the reduction in number of flares and bDMARD dose when implementing the model as a decision aid during bDMARD tapering in the DRESS trial.

**Results:**

Data from 279 bDMARD courses were used for model development. The final model included two latent DAS28-trajectories, bDMARD type and dose, disease duration, and seropositivity. The area under the curve of the final model was 0.76 (0.69–0.83) in cross-validation and 0.68 (0.62–0.73) in external validation. In simulation of prediction-aided decisions, the mean number of flares over 18 months decreased from 1.21 (0.99–1.43) to 0.75 (0.54–0.96). The reduction in he bDMARD dose was mostly maintained, increasing from 54 to 64% of full dose.

**Conclusions:**

We developed a dynamic flare prediction model, exclusively based on data typically available in routine care. Our results show that using this model to aid decisions during bDMARD tapering may significantly reduce the number of flares while maintaining most of the bDMARD dose reduction.

**Trial registration:**

The clinical impact of the prediction model is currently under investigation in the PATIO randomized controlled trial (Dutch Trial Register number NL9798).

**Supplementary Information:**

The online version contains supplementary material available at 10.1186/s13075-022-02751-8.

## Background

Many rheumatoid arthritis (RA) patients who are treated with biological disease-modifying anti-rheumatic drugs (bDMARDs) achieve long periods of low disease activity or remission [1]. However, bDMARDs may also lead to adverse events, call for self-injections or hospital visits, and are expensive [2–4]. Thus, tapering bDMARDs to the lowest effective dose is of great clinical interest and may support the sustainability of the healthcare system as a whole.

The guidelines of the European League against Rheumatism (EULAR) on the management of RA advise to consider tapering in patients that are in persistent remission [5]. In addition, numerous clinical trials and reviews provide supportive evidence to also consider tapering in patients with stable low disease activity (LDA) [6, 7]. This is in line with routine clinical practice, as maintaining a satisfactory low level of disease activity with a reduced medication dose is also of value.

The most successful and cost-effective strategy for tapering appears to be “disease activity-guided dose optimization” (DGDO) [8–10]. This means the dose is gradually tapered (usually by increasing the administration interval), until either disease activity flares or the bDMARD is discontinued. Two randomized trials have demonstrated that, using this strategy, 63–80% of patients can taper or even stop their bDMARD [8, 9]. No important difference was observed in the proportion of patients with LDA or remission after 18 months between DGDO and usual care.

However, since DGDO is a “trial and error” approach, flares occur frequently during the tapering process. In the case of a flare, the previously effective dose needs to be reinstated or additional therapy is necessary. Although these short-lived flares do not seem to relevantly affect radiographic progression or long-term disease activity, there is conflicting evidence regarding functional outcome and impact on quality of life [9, 11]. Therefore, it would be beneficial to predict whether, and to which extent, a bDMARD can be tapered in a particular patient without a flare occurring.

Several predictors for successful dose reduction or discontinuation of bDMARDs have been explored [12, 13]. However, these studies only included “baseline predictors” from before the start of the tapering process, and the strength of the evidence for these predictors is limited. Furthermore, “successful tapering” is often defined as reaching a lower bDMARD dose at some time point after the start of tapering, regardless of whether a flare occurred during the tapering process.

Therefore, this study aims to predict the likelihood of a flare occurring during bDMARD tapering at each consecutive dose reduction step. Such a dynamic prediction may be used to optimize the DGDO strategy for bDMARDs for an individual patient, as the decision for a further tapering step can be based on the predicted risk of a flare. This could minimize the number of flares during tapering, while retaining most of the bDMARD dose reduction. To facilitate future implementation of this approach in routine practice, we decided to exclusively use information easily obtainable in regular care.

## Methods

### Data extraction and preparation

#### EHR data for model development

For the development of the prediction model, electronic health record (EHR) data of two rheumatology clinics in the Netherlands were extracted for the period 2012–2019 and 2013–2019 respectively: the University Medical Center Utrecht (UMCU; an academic hospital) and Reumazorg Zuid West Nederland (RZWN; a non-academic treatment center for rheumatic diseases). In both centers, bDMARD tapering is regularly performed, but not yet standard practice. Data were extracted for all RA patients (based on ICD-10 codes) starting a bDMARD and reaching a Disease Activity Score assessing 28 joints (DAS28) < 3.2, i.e., LDA, after at least 24 weeks of treatment. The following bDMARDs were included: infliximab, adalimumab, etanercept, golimumab, certolizumab, tocilizumab, sarilumab, and abatacept. We selected patients with at least the following information available: bDMARD type and dose, seropositivity, disease duration, and ≥ 2 DAS28 measurements per year available. In addition, we aimed to extract the following data: age, gender, body mass index, concurrent and previous DMARD and glucocorticoid use, smoking status, and erosive disease.

To handle missing individual DAS28 components, we used all validated DAS28 formulae by calculating the mean of the 3- and 4-variable DAS28 formulae using ESR (erythrocyte sedimentation rate) as well as CRP (C-reactive protein) [14]. We allowed a 4-week time window between components. Flares were defined using a validated criterion: an increase in DAS28 > 1.2 compared to the previous visit, or an increase of 0.6 with a resulting DAS28 > 3.2 [15]. In addition, an “increase in bDMARD dose” was also considered a flare, to also capture flares if insufficient information was present to calculate the DAS28.

All data was extracted according to current ethical and privacy regulations in the specific hospitals. The Medical Research Ethics Committee Utrecht waived the need for informed consent, as the development data was already collected in routine care and was pseudonymized before analysis.

### DRESS data for external validation

For external validation, we extracted data from the DRESS trial [9]. In this trial, RA patients with stable LDA or remission using adalimumab or etanercept were randomized to either DGDO (*n* = 121) or routine care (*n* = 59) and followed for 18 months. The study was performed between 2011 and 2014 in two Dutch clinics (Sint Maartenskliniek Nijmegen and Woerden). The DGDO group tapered the bDMARD in three steps by increasing the administration interval every 3 months, followed by discontinuation after 6 months as long as the patient did not flare. In case of flare, the last effective dose was reinstated, and no further dose reduction attempts were undertaken. If nevertheless flares persisted, the bDMARD dose was increased to the full dose and thereafter treatment was at the rheumatologists’ discretion. In DRESS, flares were defined by the DAS28-CRP increase from baseline values.

The DRESS study (Dose REduction Strategy of Subcutaneous TNF inhibitors) was approved by the local ethics committee (Committee on Research Involving Human Subjects region Arnhem-Nijmegen), and informed consent was signed by all included patients [9].

### Model development

We developed a dynamic model to repeatedly predict the risk of a flare occurring in the next 3 months. This corresponds to a routine outpatient visit interval [13]. The model was developed using joint latent class mixed modeling, which combines a linear mixed effects- and a time-to-event model (R-package lcmm). Details of joint latent class models have been described elsewhere [16, 17].

First, in the linear mixed effects part of the model, the course (“trajectories”) of the DAS28 values over time are modeled for each patient. This is done by categorizing these trajectories into a number of subgroups: latent classes. The general form of these trajectories is defined using polynomials for the time variable. We explored models with a random intercept using 1–3 latent classes and 1st to 3rd order polynomials for the time variable, using a random slope for time. The best fitting model was selecting based on the lowest Bayesian Information Criterion (BIC) [18]. Based on the final model, each individual patient has its own predicted DAS28 trajectory.

Next, these DAS28-trajectories are used as variables in the time-to-event part of the model. The time-to-event part of the model also incorporates other variables. We explored all variables as mentioned above in “*EHR data for model development*” and selected those that had sufficient data to be extracted from the EHR. The time-to-event model was developed stepwise starting with a full model, excluding variables one by one to arrive at a final model. The decision to exclude a variable was based on clinical rationale, data availability, and improvement in model fit in cross-validation, defined by the decrease in the BIC. In short, to make individual predictions, an estimation is made about the individuals trajectory of the DAS28 over time. This trajectory is then combined with additional variables to calculate the probability of a flare occurring in the next 3 months.

We adhered to the Transparent Reporting of a Multivariable Prediction Model for Individual Prognosis or Diagnosis (TRIPOD) reporting guideline [19].

### Model validation

We assessed the accuracy of 3-monthly flare predictions in the development data with 5-fold cross-validation, using all visits at which a DAS28 was available. The area under the curve of the receiver operating characteristic (AUC-ROC) was calculated over all time-points. Other performance indicators were assessed based on an optimal cutoff as defined by Youden’s Index in the development data [15]. This index is a summary measure for sensitivity and specificity.

External validation was performed by assessing the accuracy of flare predictions in data from the DRESS trial [9]. The AUC-ROC and other performance indicators were calculated using the optimal cutoff points as determined in the development data and in DRESS data, both defined by Youden’s Index [18].

### Simulation of prediction-aided treatment

To evaluate the clinical utility of the flare predictions, we assessed the model’s potential impact on the number of flares and on the bDMARD dose used over 18 months. We simulated a new tapering strategy where the model’s predictions were used as a decision aid in the DGDO arm of the DRESS trial. At every 3-monthly visit, the predicted risk of a flare was taken into account when deciding to continue or to stop tapering. The predicted risk of flare was categorized into a high predicted risk (above or equal to the optimal cutoff point), or a low predicted risk (below the cutoff point). The simulation was based on the following assumptions:

1. If a flare occurred in the DRESS trial before the model predicted a high risk of flare, this flare also occurs in the simulation. The bDMARD dose is the same as in the trial. Thus, there is no impact of the predictions is observed in this case.
2. If the model predicted a high risk of flare in simulation and no flare had occurred in the DRESS trial thus far, the bDMARD is not tapered further (kept at a constant dose). No flares occur in simulation during the remaining follow-up, except for the scenario described in 4.
3. If a patient had completely discontinued the bDMARD in the DRESS trial when the model predicted a high predicted risk of flare, the bDMARD dose in simulation is increased to and kept at 50% of the full registered dose. This corresponds to the last tapering step. No flares occur during the remaining follow-up, except for the scenario described in 4.
4. If in the DRESS trial a flare occurred after the model predicted a high risk of flare and the bDMARD dose in DRESS was equal to or higher than the bDMARD dose in simulation, that flare also occurs in the simulation. The bDMARD dose is equal to the DRESS trial during the remaining follow-up.

The number of flares occurring, the proportion of patients experiencing at least one flare, and the proportion of the full registered dose were calculated. These were then compared between the simulation and the DGDO arm of the DRESS trial over 18 months. Confidence intervals (CI) were calculated using 1000-fold bootstrapping. As there is no obvious optimum in the trade-off between the reduction in the number of flares and the increase in bDMARD dose, we evaluated the clinical impact of prediction-aided treatment for several cutoffs around the optimal cutoffs as defined by Youden’s Index [18].

## Results

### Patient characteristics

**Fig. 1:**
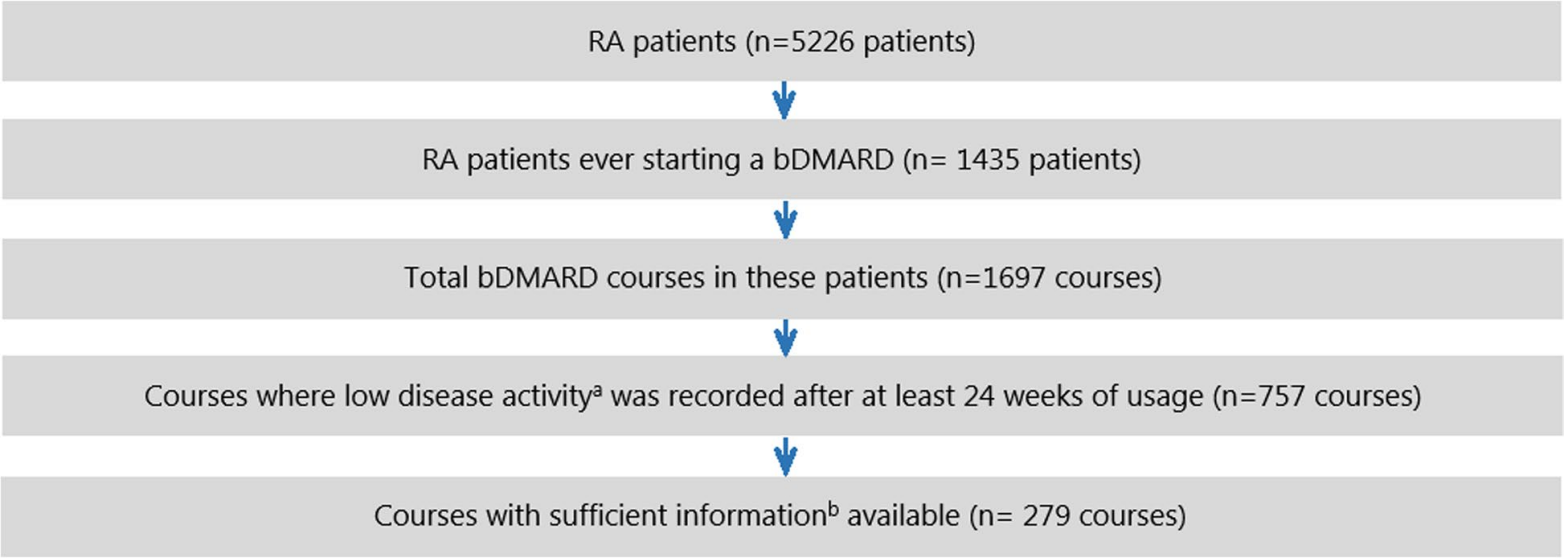
Selection of bDMARD courses from EHR data for model development. **a** Low disease activity was defined as a DAS28 (ESR or CRP) ≤ 3.2. **b** Based on the availability of at least two DAS28 measurements per year, bDMARD type and dose, disease duration, and seropositivity. bDMARD, biological disease*-*modifying antirheumatic drug; EHR, electronic health record; RA, rheumatoid arthritis

Of the total number of 5226 RA patients in the EHR data, there were 757 bDMARD courses in which LDA was recorded after at least 24 weeks of usage (Fig. 1). In 279 bDMARD courses of 255 patients, sufficient data was available for model development (see the “Methods” section). Data for smoking, erosive disease, concurrent and previous DMARDs, and glucocorticoids were of insufficient quality (> 50% missing data) and/or could not be (easily) extracted from the EHR. The median follow-up time of the included bDMARD courses was 21 months, and the mean bDMARD dose was 76.7% of the full dose. Table 1 displays general patient characteristics of the development data and the data from the DRESS trial used for external validation. Significant differences between the populations were observed for age, DAS28 at baseline, the number of DAS28-measurements, flare rate, and bDMARD dose, among others.

**Table 1:** Patient characteristics in data for model development and external validation

Characteristics	Development data (***n*** = 279)	DRESS data for external validation (***n*** = 164)	***P***-value of difference^**a**^
**General characteristics**			
Age, mean in years (SD)	50.2 (17.1)	58.7 (9.9)	< 0.01
Female, *N* (%)	203 (72.8%)	105 (64%)	0.05
BMI, median in kg/m^2^ (IQR)	25.4 (22.6–30.0)	26.8 (23.3–29.5)	0.22
Height, mean in cm (SD)	170.4 (12.5)	172 (9.2)	0.15
Weight, mean in kg (SD)	77.0 (17.2)	78.8 (15.5)	0.27
Follow-up time, median in months (SD)	21 (2.0)	18.7 (1.6)	< 0.01
**RA characteristics**			
Disease duration at start of bDMARD, median in years (IQR)	9.0 (5.0–16.5)	6.0 (2.7–12.7)	0.38
Positivity for RF and/or ACPA, *N* (%)	236 (84.6%)	140 (85.4%)	0.83
**Biological**			
bDMARD type, *N* (%)			
Etanercept	77 (27.6%)	107 (65.2 %)	< 0.01
Infliximab	5 (1.8%)	–	–
Adalimumab	113 (40.5%)	57(34.8%)	0.23
Certolizumab	10 (3.6%)	–	–
Golimumab	18 (6.5%)	–	–
Tocilizumab	8 (2.9%)	–	–
Sarilumab	2 (0.7%)	–	–
Abatacept	37 (13.3%)	–	–
Time from start bDMARD baseline, mean in weeks (SD)^b^	10 (7.7)	42.1 (29.1)	< 0.01
Prescribed dose during follow-up (mean, expressed as % of full dose)	76.7%	61.6%	< 0.01
**Disease activity**			
DAS28 at baseline, mean (SD)	2.79 (1.34)	2.15 (0.70)	< 0.01
VAS GH at baseline, median (IQR)	30 (11–40)	20 (10–34)	0.76
TJC at baseline, median (IQR)	0 (0–1)	0 (0–1)	–
SJC at baseline, median (IQR)	0 (0–1)	0 (0–1)	–
ESR at baseline, median in mm/hour (IQR)	7 (3–12)	13 (7–22)	< 0.01
CRP at baseline, median mg/ml (IQR)	2.7 (1.3–5.0)	3 (3–3)	0.22
Increase in TJC (yes/no)^c^, *N* (%)	49 (17.6%)	NA	–
Increase in SJC (yes/no)^c^, *N* (%)	28 (10.0%)	NA	–
No. of DAS28 measurements, mean (SD)	6.1 (3.8)	7.3 (1.2)	< 0.01
Time between DAS28, mean in weeks (SD)	22.3 (12.3)	12.0 (5.4)	< 0.01
Flare rate (# flares per patient year)	0.47	0.62	< 0.01
DAS28 measurement rate (#DAS28 measurements per patient year)	2.18	4.72	0.04

*ACPA* anti-citrullinated protein antibodies, *bDMARD* biological disease-modifying antirheumatic drug, *CRP* C-reactive protein, *DAS28* disease activity score based on 28 joint count, *EHR* electronic health record, *ESR* erythrocyte sedimentation rate, *IQR* interquartile range, *RF* rheumatoid factor, *SD* standard deviation, *TJ(C)/SJ(C)* tender/swollen joint count, *VAS GH* an assessment of general health on a visual analog scale (0–100 mm)

^a^*P*-values based on *T*-test for normally distributed continuous data, Mann-Whitney *U* test for continuous non-normally distributed data, and *χ*^2^ test for nominal data

^b^In the development data, baseline is defined as the first DAS28 ≤ 3.2

^c^An increase in TJC and/or SJC (yes/no) at baseline relative to the previous TJC/SJC measurement

### Model development

**Fig. 2:**
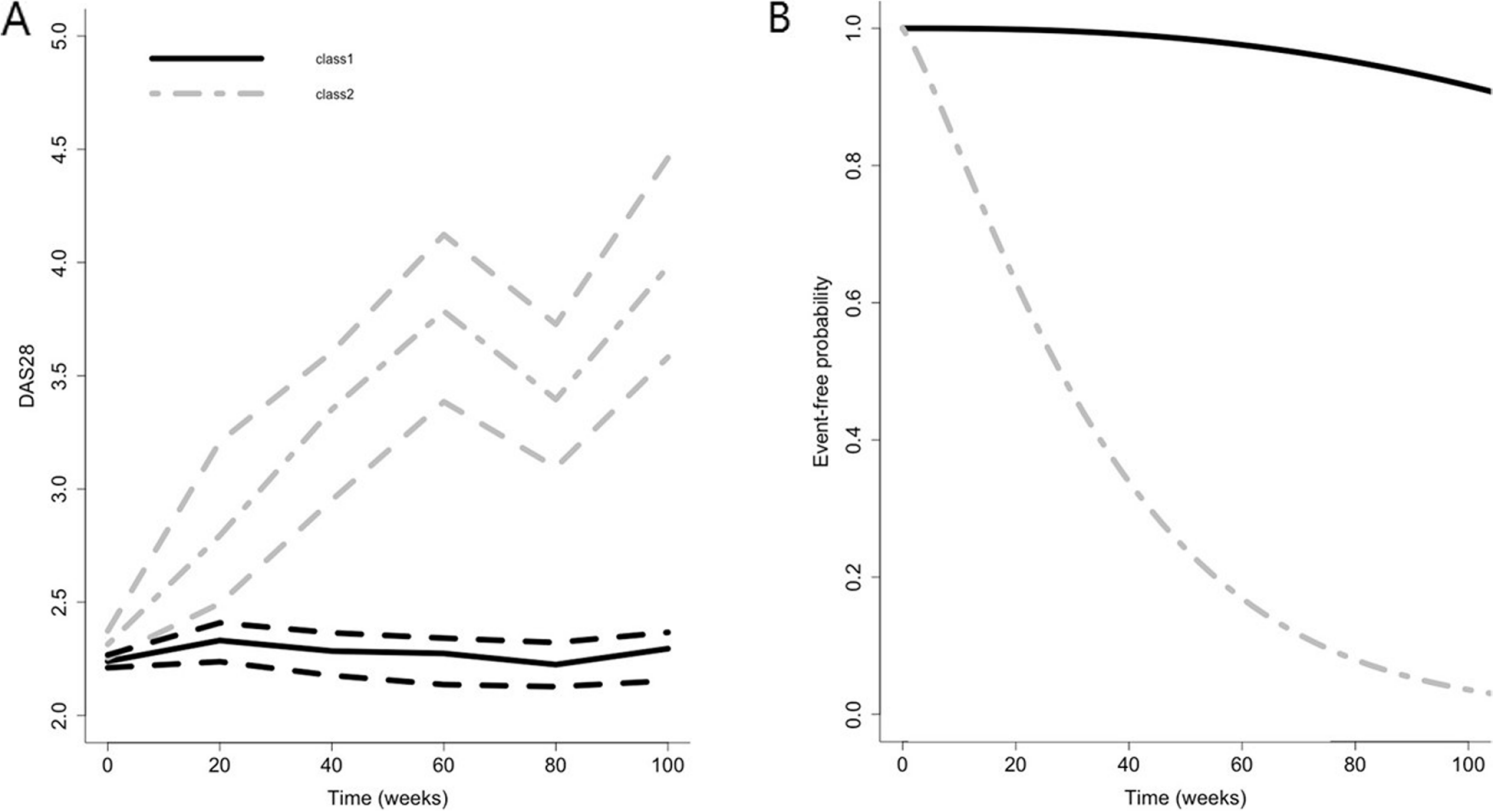
Mean DAS28-trajectories of identified latent classes and their relation to the occurrence of a flare. **A** The mean course of the disease activity score (DAS28) over time in patients assigned to one of the two “latent trajectory classes.” In class 1 (*n* = 182), a stable low disease activity is observed, whereas patients in class 2 (*n* = 97) display an increasing disease activity over time. **B** The probability of remaining free from flares over time for patients assigned to one the “latent trajectory classes” for disease activity, as displayed on the left. Patients in class 2 display a shorter time to flare as compared to patients in class 1

The variables that were retained in the final prediction model and the corresponding hazard ratios are displayed in Table 2. The final model identified two latent DAS28-trajectories, defined by a linear and a quadratic time coefficient. Figure 2 shows the mean of these two trajectories (left), together with their respective time to flare (right). The course of disease activity in the class 2 DAS28-trajectory shows an increase in disease activity over time and a shorter time to flare, compared to the class 1 DAS28-trajectory. Variables that significantly increased the likelihood of a flare were seropositivity, bDMARD dose < 50% and an increase in tender joint count at baseline (compared to previous visit).

**Table 2:** Variables of the final flare prediction model

Parameter	Hazard ratio (95% CI)
Linear time coefficient DAS28 trajectory latent class 1	1.04 (1.02–1.06)
Quadratic time coefficient DAS28 trajectory latent class 1	1.66 (0.42–6.55)
Linear time coefficient DAS28 trajectory latent class 2	1.14 (1.08–1.20)
Quadratic time coefficient DAS28 trajectory latent class 2	4.52 (3.83–5.33)
Time to reach stable low disease activity (weeks)^a^	0.97 (0.96–0.98)
DAS28 at baseline	1.18 (0.90–1.54)
Prescribed dose (% of standard dose) at baseline	1.21 (0.88–1.67)
SJ increase at baseline (yes/no)^b^	1.72 (0.94–3.17)
TJ increase at baseline (yes/no) ^b^	2.07 (1.13–3.81)
Disease duration (years) at start of bDMARD	1.02 (0.99–1.05)
Seropositivity (RF and/or ACPA)	2.51 (1.39–4.53)
bDMARD TNFi type (yes/no)	0.90 (0.54–1.49)
bDMARD dose ≤50% of full registered dose (time-varying variable)	2.21 (1.73–2.82)

*ACPA* anti-citrullinated protein antibody, *bDMARD* biological disease-modifying antirheumatic drug, *DAS28* disease activity score based on 28-joint count, *RF* rheumatoid factor, *TJ(C)/SJ(C)* tender/swollen joint count, *TNFi* tumor necrosis factor inhibitor

In development data, baseline is defined as the first DAS28 ≤ 3.2 (low disease activity)

^a^In development data: time from start biological until DAS28 < 3.2 for the first time. In DRESS data: time from start biological until baseline visit

^b^An increase in TJC or SJC (yes/no) at baseline, compared to the previous DAS28 measurement

As the DAS28-trajectories represent the course of disease activity over time, this is a time-dependent variable. By default, only one continuous time-dependent variable can be included in a joint latent class model. In order to also add bDMARD dose as a second time-dependent variable, it was dichotomized in < or ≥ 50% of the full registered dose.

### Model validation

Predictive performance in cross-validation and external validation is summarized in Table 3. In cross-validation, the model achieved an AUC-ROC of 0.76 (CI 0.69–0.83). The optimal cutoff in the development data was at a predicted probability of flare of 14.3% within the next 3 months. For external validation, sixteen patients were excluded from DRESS because of missing predictor information. Furthermore, as the variable “increase in SJC/TJC” was not available in the DRESS data, these were set to 0 (i.e. no increase) for the validation, with the rationale that patients that met the DRESS inclusion criteria had a stable low level of disease activity at baseline. Supplementary Figure S1 shows the AUC-ROC of the model in external validation (0.68 (CI 0.62–0.73), see Supplementary Figure S2 for the calibration plot). The optimal cutoff point in DRESS data was found to be at a predicted chance of flare of 31.5% within the next 3 months.

**Table 3:** Predictive performance in cross validation and external validation

	Cross validation (cutoff 14.3%)	External validation (cutoff 14.3%)	External validation (cutoff 31.5%)
AUC	0.76 (0.69–0.83)	0.68 (0.62–0.73)	0.68 (0.62–0.73)
Sensitivity (%)	86.1 (81.9–90.1)	73.2 (64.4–82.0)	58.8 (49.0–68.6)
Specificity (%)	66.5 (60.1–72.5)	52.0 (0.48–56.0)	68.7 (64.9–72.4)
Positive predictive value (%)	33.0 (29.3–38.5)	20.1 (15.9–24.3)	23.7 (18.3–29.0)
Negative predictive value (%)	96.2 (95.4–98.4)	92.1 (89.2–95.0)	91.0 (88.3–93.6)
Accuracy (%)	70.6 (65.6–75.6)	55.0 (51.2–58.7)	67.3 (63.6–70.8)

Results from the 5-fold cross-validation in development data are presented for an optimal cutoff point of 14.3% as determined with Youden’s index. The results from external validation in the DRESS trial [9] are presented for 2 different cutoff points: the optimal cutoff point from the development data (14.3%), and the optimal cutoff point in the DRESS data as determined by Youden’s index (31.5%). 95% confidence intervals are presented between brackets

*AUC* area under the curve

Because the model cannot truly function as a “joint” model at baseline, since no longitudinal information is yet available, we also explored the performance when removing baseline predictions. This indeed improved the AUC in external validation to 0.71 (CI 0.64-0.77, Supplementary Figure S3 and Supplementary Table S1).

### Simulation of prediction-aided treatment

We assessed the potential clinical impact of the model on the number of flares and the amount of bDMARD dose reduction, when used as a decision aid within a DGDO strategy. The clinical impact of prediction-aided treatment in simulation was evaluated for cutoffs from 15–45% in steps of 10% (Supplementary Table S2), and results were discussed to determine the optimal cutoff for clinical practice. A risk cutoff of 35% was deemed optimal, as this significantly reduced the number of flares per patient over 18 months from 1.21 (0.99–1.43) to 0.75 (0.54–0.96), while retaining most of the bDMARD dose reduction (64% vs 54% of full registered dose used). See Table 4. When using this optimal cutoff of 35%, only 1.0 flare occurred for each full dose that was tapered in the simulation of prediction-aided treatment, versus 2.0 flares in the DRESS DGDO arm. Furthermore, in the DRESS routine care arm, each prevented flare (compared with DRESS DGDO) came at a cost of 51% of a full bDMARD dose over 18 months, while this was only 22% in the simulated prediction-aided group. As the AUC-ROC improved when the predictions at baseline were not taken into account, we explored the simulation of prediction-aided treatment when removing the baseline predictions. However, the simulation results were hardly influenced by this (Supplementary Table S3).

**Table 4:** Flares and bDMARD dose in simulation of prediction-aided treatment

	DRESS routine care	Simulation (cutoff: 35%)	DRESS DGDO
Mean no. of flares (95% CI)	0.48 (0.24–0.72)	0.75 (0.54–0.96)	1.21 (0.99–1.43)
Decrease in flares compared to DRESS DGDO (95% CI)	0.73 (0.40–1.0)	0.46 (0.16–0.74)	–
Mean bDMARD dose (95% CI)	0.91 (0.86–0.96)	0.64 (0.61–0.68)	0.54 (0.50–0.58)
Increase in bDMARD dose compared to DRESS DGDO (95% CI)	0.37 (0.31–0.44)	0.10 (0.05–0.16)	–
Percentage of patients flaring (95% CI)	27% (15–40)	45% (36–54)	71% (63–79)
Increase in bDMARD dose per flare prevented vs. DRESS DGDO^a^ (95% CI)	0.51 (0.44–0.59)	0.22 (0.15–0.32)	–
Number of extra flares per full bDMARD dose saved vs. routine care^b^ (95% CI)	–	1.0 (0.3–1.8)	2.0 (1.4–2.6)

*bDMARD* biological disease*-*modifying antirheumatic drug, *DGDO* disease activity-guided dose optimisation

^a^The difference in mean bDMARD dose divided by the difference in mean flares compared with DRESS [9] DGDO. This represents the increase in bDMARD dose that was needed to prevent a flare over 18 months for this tapering strategy

^b^The mean difference in the number of flares, divided by the mean difference in bDMARD dose, compared to routine care. This represents the number of extra flares that occurred for each full dose of bDMARD that is tapered compared to routine care over 18 monhts using this tapering strategy

## Discussion

The goal of this study was to develop and validate a flare prediction model to reduce the number of flares during bDMARD tapering, exclusively using data that can easily be obtained in routine care. Our simulation results show that the addition of our flare prediction model to a DGDO tapering strategy is both superior to routine care and to DGDO alone, when considering the ratio between the number of flares and amount of bDMARD dose reduction. To our knowledge, this is the first study not only developing a dynamic flare prediction model, but also performing an external validation and subsequent simulation of clinical impact in the context of bDMARD tapering.

As tapering bDMARDs is of great clinical interest, other studies have also investigated predictors in the context of tapering. Several studies and systematic reviews have investigated the predictive value of biomarkers, serum drug levels, or PET-scans during bDMARD tapering [12, 20–22]. However, none of these studies showed a clear predictive value of these markers. In addition, the study by Verhoef et al. showed that for a biomarker to be cost-effective during bDMARD tapering, it must be inexpensive and have high sensitivity and specificity [23]. If future studies do show a predictive value of (bio)markers during tapering, these can be included in the prediction model. The added predictive value of such markers and their cost-effectiveness should then be assessed. An important advantage of the current model is that it only includes variables that are routinely collected in RA clinical practice, thereby enhancing feasibility and cost-effectiveness.

A recent review [13] focused on predictors for successful discontinuation, rather than tapering, of bDMARDs. Similar to the current study, they found seropositivity, LDA, disease duration, and CRP/ESR to be possible predictors of value. In addition, they mention physical functioning and ultrasound measures as possible predictors. However, the studies included in this review were often small and too heterogeneous to compare in meta-analysis. Furthermore, only fixed baseline variables were included, rather than performing dynamic predictions using information over time.

Two studies have incorporated such dynamic variables to predict RA disease activity over time [24, 25]. The study by Norgeot et al. [24] found the Clinical Disease Activity Index (CDAI), CRP/ESR, glucocorticoid use, and other DMARD use to be important predictors. However, this study is not performed in the specific context of tapering bDMARDs. The model developed by Vodenčarević et al. [25] does focus specifically on bDMARD tapering. However, this model is developed and validated on the clinical trial data of 41 patients only and may therefore be difficult to extrapolate to routine care. Both of these dynamic prediction models were developed using machine learning techniques. We have previously also explored the potential of a machine learning model similar to Vodenčarević et al. [26]. However, we chose to pursue the joint latent class model as the performance was similar, and the joint latent class model is more transparent regarding the DAS28-trajectories used and the effects of covariates in the model (i.e., providing hazard ratios).

A major unique strength of this study is that the model’s performance is assessed in external validation. There were several significant differences between the patient populations from routine care used for developing the model and the DRESS pragmatic trial data for external validation regarding baseline characteristics, disease activity, and bDMARD treatment. However, despite these differences the model retained an adequate performance in the external validation, indicating that these differences do not invalidate the model. Another strength is that the clinical impact is evaluated in simulation. In this simulation, successful tapering was not only defined by reaching a lower bDMARD dose, but also by the number of flares during tapering. Furthermore, our model was developed using easily obtainable parameters from routine care EHR data, rather than, e.g., clinical trial data or specific biomarkers [27].

The AUC in cross-validation and external validation (0.76 and 0.68, respectively) may be interpreted as only a moderate performance. However, the AUC may not be the most suitable measure to assess the model’s clinical utility. The added value in clinical practice is determined by the effects of prediction-aided treatment on the rate of flares and the amount of bDMARD dose reduction, when compared to the available alternatives. The currently existing alternatives are either continuing the bDMARD at full dose or tapering until a flare occurs in a trial-and-error approach. Our simulation results show that prediction-aided treatment is superior to both these alternatives regarding the ratio between the number of flares and the amount of bDMARD dose reduction. Therefore, prediction-aided treatment may present the best available bDMARD tapering strategy. This is currently being investigated in the PATIO randomized controlled clinical trial (Dutch Trial Register number NL9798).

Interestingly, the AUC of the prediction model improved in external validation from 0.68 to 0.71 when baseline predictions were removed. This is likely because the model can only function as a “joint” model when longitudinal information is available. This effect on AUC was also observed in the development data, but due to the relative overrepresentation of baseline visits in the DRESS data compared to the development data, this was less pronounced. As the removal of baseline predictions had almost no effect on the simulation of clinical impact, we chose to retain these predictions. Including disease activity measures prior to the start of tapering could potentially improve the performance of our model, as this would ensure that longitudinal information is available at baseline.

A challenge in this study was the limited data quality regarding the frequency of DAS28 measurements in the development data. This might also have contributed to the different flare rates and resulting discrepancy between the optimal cutoff points in the development data and external validation data from the DRESS trial. When implementing a prediction-aided bDMARD tapering strategy in clinical practice or clinical studies, a treat-to-target (T2T) strategy with regular (e.g., 3 monthly) DAS28 measurements should be used, in line with EULAR recommendations [5]. As the DAS28 measurement frequency in the DRESS trial best reflects these recommendations, the optimal cutoff point found in simulation (i.e. 35%) is likely the most suitable for implementation of the model in clinical practice.

Besides the DAS28 measurements, several other parameters were also difficult to extract as structured data from the EHR, such as smoking, concurrent csDMARDs, and erosiveness of disease. We explored imputation to increase the amount of these data points, but this did not improve the model’s performance in cross-validation. Improved registration of these parameters and the optimization of free text mining techniques could allow for future inclusion of these parameters in model development and possibly a better performance. Importantly, the results from external validation are not biased by missing data, since the DRESS data had a standard measurement frequency and very few data missing on disease activity. Therefore, we think our simulation should be an accurate representation of the potential clinical impact of using the models predictions as an decision aid added to a DGDO strategy.

Since prediction-aided treatment could reduce the number of flares during bDMARD tapering, patients and physicians may be more willing to start tapering with such a prediction model than without [28]. Furthermore, our prediction model can be used as an add-on to DGDO, retains most of the bDMARD reduction as attained by DGDO, and is a low cost intervention. Therefore, the model might prove to be an even more cost-effective strategy than DGDO alone [10]. The clinical implementation may be relatively straightforward, as it uses only predictors usually available in the EHR.

## Conclusions

In conclusion, we developed and validated a dynamic prediction model to predict the risk of a flare occurring within 3 months during a bDMARD tapering strategy. In simulation, we showed that a prediction-aided treatment strategy has the potential to significantly reduce the number of flares, while maintaining most of the bDMARD dose reduction. As this simulation is inevitably based on certain assumptions, we are currently investigating the clinical impact of prediction aided treatment in the PATIO randomized controlled trial. The current study and the PATIO-trial provide the next step towards the successful implementation of personalized medicine using clinical decision support systems.

## Supplementary Information

**Additional file 1: Supplementary Figure S1.** Receiver operating characteristic (ROC) curve in external validation. ROC-curve of the model in external validation in data of the Dose Reduction Strategy of Subcutaneous TNF inhibitors (DRESS) trial [9].

**Additional file 2: Supplementary Figure S2.** Calibration plot of flare prediction model including baseline predictions Calibration plot in external DRESS-data [9]. Patients were grouped based on their predicted probability from lowest to highest predicted 3-monthly risk of flare (x-axis) using the median, 25th and 75th percentile. On the y-axis these groups are compared with the observed frequency of flare within 3 months. Perfectly calibrated predictions would be expected to be at the diagonal.

**Additional file 3: Supplementary Figure S3.** AUC and calibration plot without baseline predictions. A. Receiver operator characteristic (ROC)-curve of external validation of the flare prediction model in DRESS data [9], where baseline predictions are removed. The rationale is that the prediction model cannot truly function as a ‘joint’ model at baseline, as no longitudinal data is available. B: Calibration plot in DRESS-data, excluding baseline predictions. Patients were grouped based on their predicted probability from lowest to highest predicted 3-monthly risk of flare (x-axis) using the median, 25th and 75th percentile. On the y-axis these groups are compared with the observed frequency of flare within 3 months. Perfectly calibrated predictions would be expected to be at the diagonal. AUC: Area Under the Curve.

**Additional file 4: Supplementary Table S1.** Predictive performance without baseline predictions in DRESS data. 95% confidence intervals are presented between brackets. The results from external validation in the DRESS trial [9] without baseline predictions. The rationale for leaving out baseline predictions is that the prediction model cannot truly function as a ‘joint’ model at baseline, as no longitudinal data is available. The results for 2 different cutoff points are presented: the optimal cutoff point from the development data (14.3%) and the optimal cutoff point in the DRESS data as determined by Youden’s index (31.5%). AUC: Area under the curve.

**Additional file 5: Supplementary Table S2.** Simulation results for different cutoff points (baseline predictions included). 95% confidence intervals are presented between brackets. a. The mean difference in bDMARD dose divided by the mean number of flares compared with the DRESS [9] DGDO arm. The number therefore represents the increase in bDMARD dose that was needed to prevent a flare for this specific tapering strategy. b. The mean difference in the number of flares, divided by the mean difference in bDMARD dose, compared to routine care. The ratio thus represents the number of extra flares that occurred for each extra full dose of bDMARD that is tapered compred to routine care over 18 monhts using this specific tapering strategy. bDMARD: biological disease-modifying antirheumatic drug, DGDO: disease activity guided dose optimisation.

**Additional file 6: Supplementary Table S3.** Simulation results with and without baseline predictions. 95% confidence intervals are presented between brackets. The results from external validation in the DRESS trial [9] without baseline predictions, for the optimal cutoffpoint of 35% as determined in simulation (see Supplementary Table S2). The rationale for leaving out baseline predictions is that the prediction model cannot truly function as a ‘joint’ model at baseline, as no longitudinal data is available. a. The mean difference in bDMARD dose divided by the mean number of flares compared with the DRESS DGDO arm. The number therefore represents the increase in bDMARD dose that was needed to prevent a flare for this specific tapering strategy. b. The mean difference in the number of flares, divided by the mean difference in bDMARD dose, compared to routine care. The ratio thus represents the number of extra flares that occurred for each extra full dose of bDMARD that is tapered compred to routine care over 18 monhts using this specific tapering strategy. bDMARD: biological disease-modifying antirheumatic drug, DGDO: disease activity guided dose optimisation.

## Data Availability

Data from the DRESS trial is available according to FAIR principles. The regular care data of UMCU / RZWN patients is not available, as these patients did not agree for their data to be shared publicly.

## Abbreviations

ACPA: Anti-citrullinated protein antibodies
AUC: Area under the curve
(b)DMARD: (Biological) disease-modifying antirheumatic drug
BIC: Bayesian Information Criterion
CI: Confidence interval
CRP: C-reactive protein
DAS28: Disease activity score based on the assessment of 28 joints
DGDO: Disease activity-guided dose optimization
DRESS: Dose REduction Strategy of Subcutaneous tumor necrosis factor inhibitors (9)
EHR: Electronic Health Record
ESR: Erythrocyte sedimentation rate
EULAR: European League against Rheumatism
IQR: Interquartile range
LDA: Low disease activity
RA: Rheumatoid arthritis
RF: Rheumatoid factor
ROC: Receiver operator characteristic
RZWN: Reumazorg Zuid West Nederland
SD: Standard deviation
SJ(C): Swollen joint (count)
TJ(C): Tender joint (count)
TNFi: Tumor necrosis factor inhibitor
UMCU: University Medical Centre Utrecht
VAS GH: An assessment of general health on a visual analog scale (0–100 mm)
