# Supplementary figures and images for: Using real-world data to dynamically predict flares during tapering of biological DMARDs in rheumatoid arthritis: development, validation, and potential impact of prediction-aided decisions
Source: Arthritis Res Ther. 2022 Mar 23;24:74. doi: 10.1186/s13075-022-02751-8 (PMC8941811; doi:10.1186/s13075-022-02751-8)

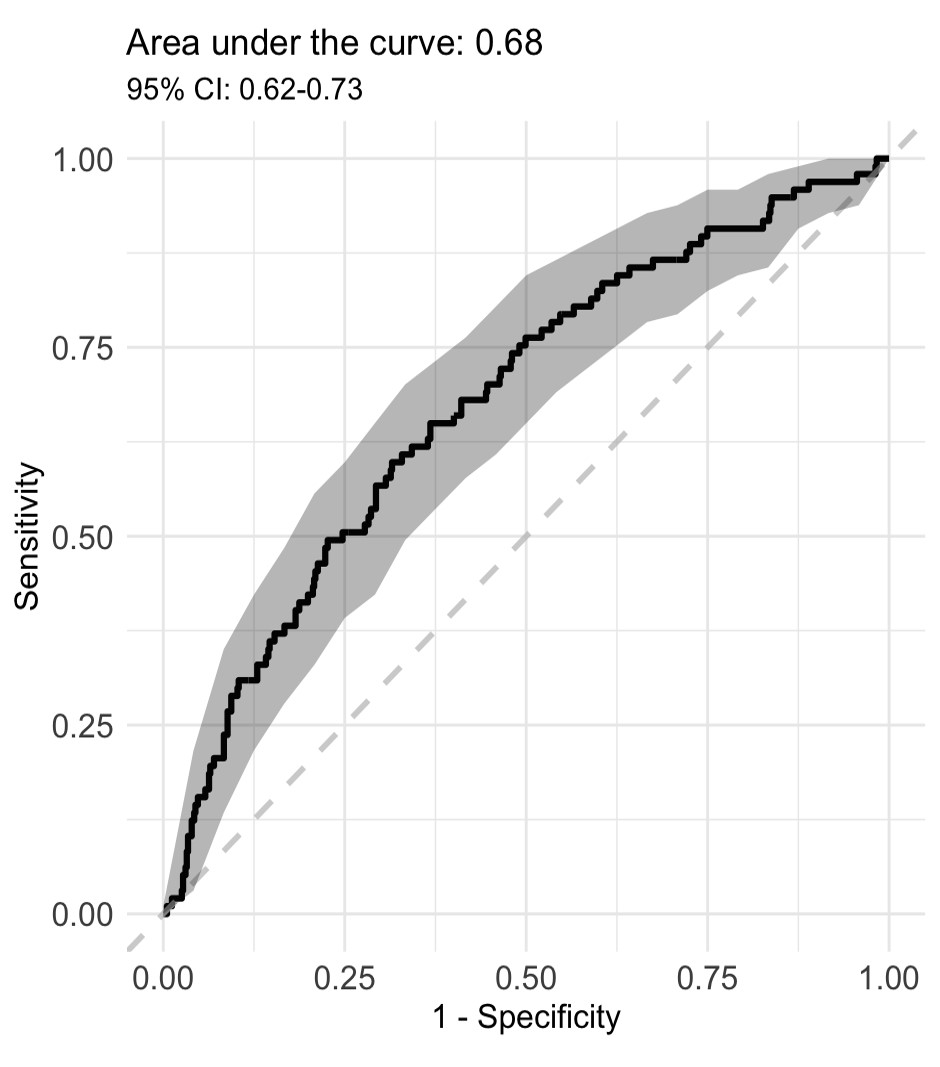

Supplement: Supplementary file 1 — Additional file 1: Supplementary Figure S1. Receiver operating characteristic (ROC) curve in external validation. ROC-curve of the model in external validation in data of the Dose Reduction Strategy of Subcutaneous TNF inhibitors (DRESS) trial [9]. [file 13075_2022_2751_MOESM1_ESM.jpg]

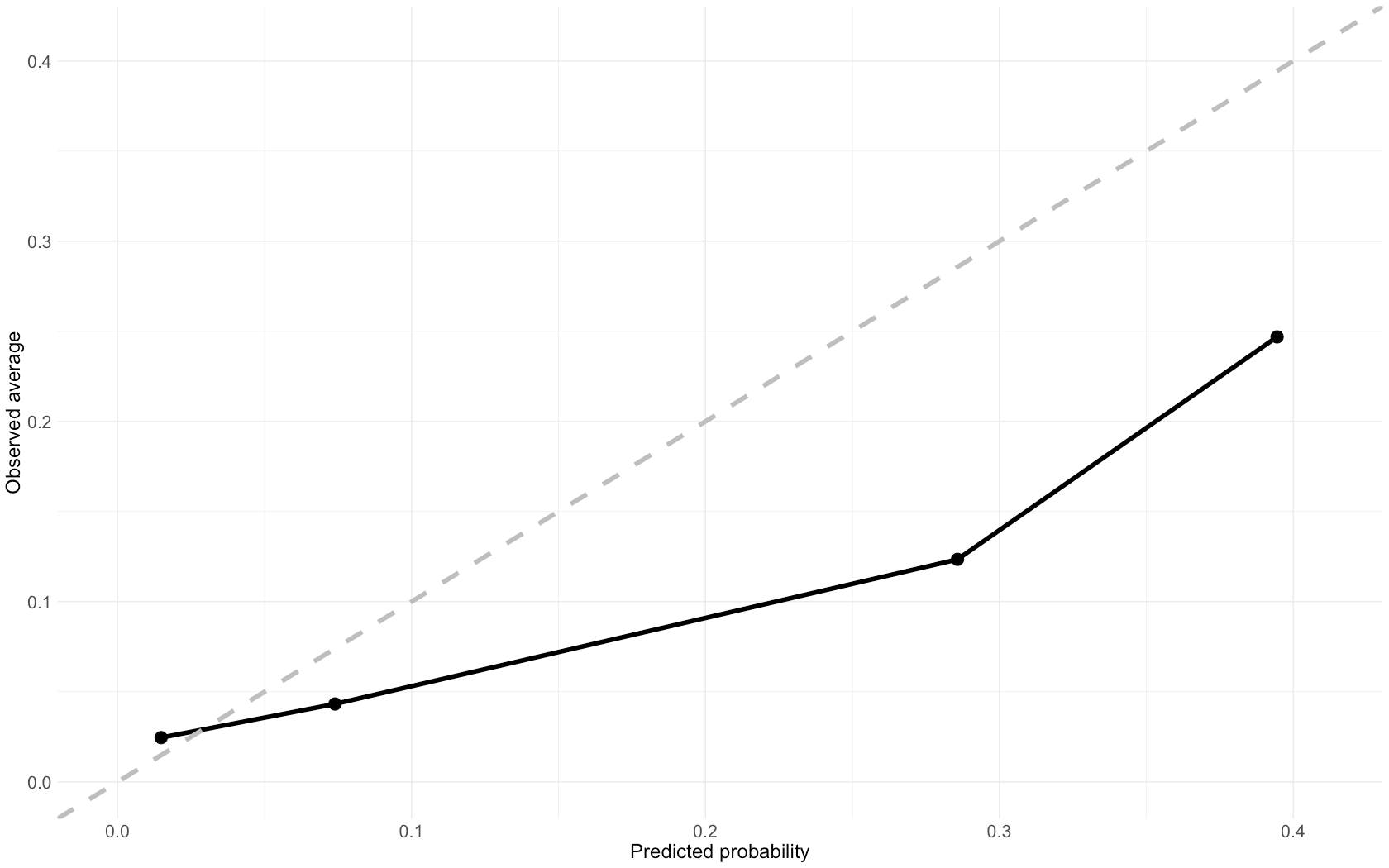

Supplement: Supplementary file 2 — Additional file 2: Supplementary Figure S2. Calibration plot of flare prediction model including baseline predictions Calibration plot in external DRESS-data [9]. Patients were grouped based on their predicted probability from lowest to highest predicted 3-monthly risk of flare (x-axis) using the median, 25th and 75th percentile. On the y-axis these groups are compared with the observed frequency of flare within 3 months. Perfectly calibrated predictions would be expected to be at the diagonal. [file 13075_2022_2751_MOESM2_ESM.png]

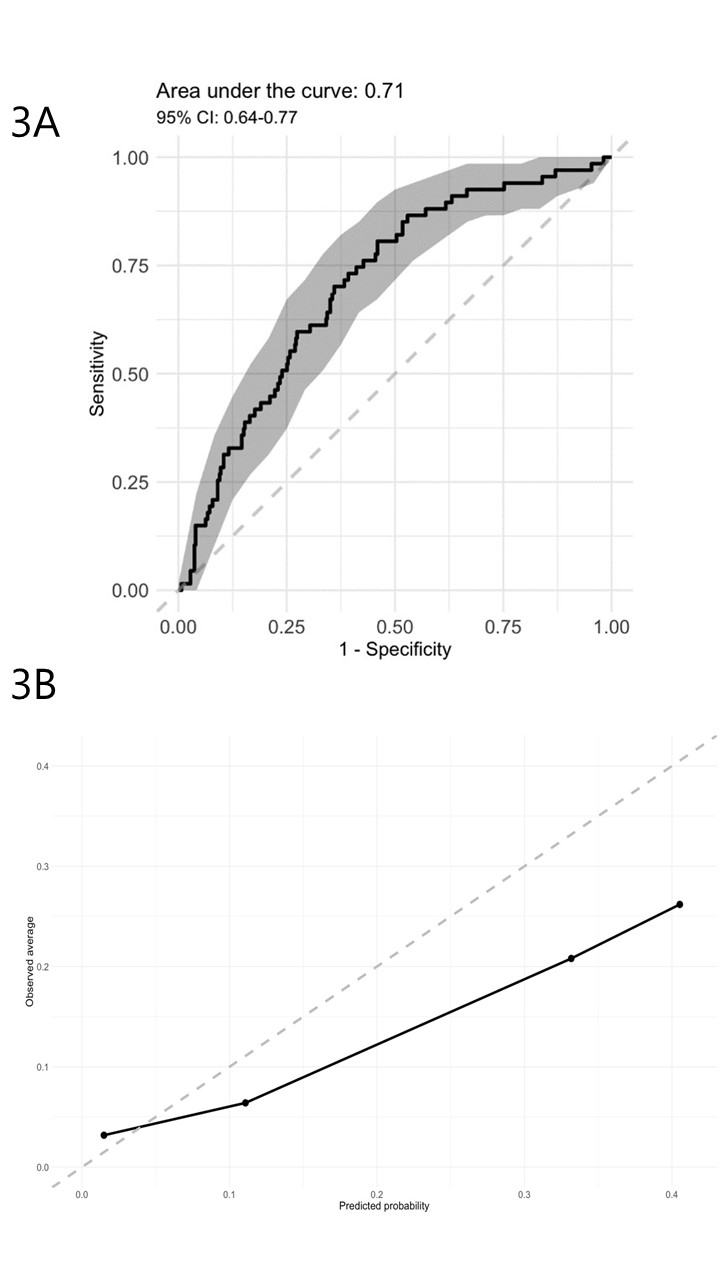

Supplement: Supplementary file 3 — Additional file 3: Supplementary Figure S3. AUC and calibration plot without baseline predictions. A. Receiver operator characteristic (ROC)-curve of external validation of the flare prediction model in DRESS data [9], where baseline predictions are removed. The rationale is that the prediction model cannot truly function as a ‘joint’ model at baseline, as no longitudinal data is available. B: Calibration plot in DRESS-data, excluding baseline predictions. Patients were grouped based on their predicted probability from lowest to highest predicted 3-monthly risk of flare (x-axis) using the median, 25th and 75th percentile. On the y-axis these groups are compared with the observed frequency of flare within 3 months. Perfectly calibrated predictions would be expected to be at the diagonal. AUC: Area Under the Curve. [file 13075_2022_2751_MOESM3_ESM.jpg]
