# Supplementary material for: Using real-world data to dynamically predict flares during tapering of biological DMARDs in rheumatoid arthritis: development, validation, and potential impact of prediction-aided decisions
Source: Arthritis Res Ther. 2022 Mar 23;24:74. doi: 10.1186/s13075-022-02751-8 (PMC8941811; doi:10.1186/s13075-022-02751-8)
Supplement: Supplementary file 4 — Additional file 4: Supplementary Table S1. Predictive performance without baseline predictions in DRESS data. 95% confidence intervals are presented between brackets. The results from external validation in the DRESS trial [9] without baseline predictions. The rationale for leaving out baseline predictions is that the prediction model cannot truly function as a ‘joint’ model at baseline, as no longitudinal data is available. The results for 2 different cutoff points are presented: the optimal cutoff point from the development data (14.3%) and the optimal cutoff point in the DRESS data as determined by Youden’s index (31.5%). AUC: Area under the curve. [file 13075_2022_2751_MOESM4_ESM.docx]

|  | External validation cut-off 14.3% | External validation cut-off 31.5% |
| --- | --- | --- |
| AUC | 0.71 (0.64–0.77) | 0.71 (0.64–0.77) |
| Sensitivity (%) | 86.6 (78.4–94.7) | 71.6 (60.8–82.4) |
| Specificity (%) | 46.9 (42.2–51.6) | 61.3 (56.7–65.9) |
| Positive Predictive Value (%) | 20.2 (15.6–24.6) | 22.3 (16.8–27.9) |
| Negative Predictive Value (%) | 95.7 (93.0-98.5) | 93.3 (90.4–96.2) |
| Accuracy (%) | 52.2 (47.7–56.7) | 62.7 (58.2–66.9) |

Supplementary Table S1: Predictive performance without baseline predictions in DRESS data
95% confidence intervals are presented between brackets. The results from external validation in the DRESS trial(9) without baseline predictions. The rationale for leaving out baseline predictions is that the prediction model cannot truly function as a ‘joint’ model at baseline, as no longitudinal data is available. The results for 2 different cut-off points are presented: the optimal cut-off point from the development data (14.3%) and the optimal cut-off point in the DRESS data as determined by Youden’s index (31.5%). AUC: Area under the curve
