# Supplementary material for: Using real-world data to dynamically predict flares during tapering of biological DMARDs in rheumatoid arthritis: development, validation, and potential impact of prediction-aided decisions
Source: Arthritis Res Ther. 2022 Mar 23;24:74. doi: 10.1186/s13075-022-02751-8 (PMC8941811; doi:10.1186/s13075-022-02751-8)
Supplement: Supplementary file 6 — Additional file 6: Supplementary Table S3. Simulation results with and without baseline predictions. 95% confidence intervals are presented between brackets. The results from external validation in the DRESS trial [9] without baseline predictions, for the optimal cutoffpoint of 35% as determined in simulation (see Supplementary Table S2). The rationale for leaving out baseline predictions is that the prediction model cannot truly function as a ‘joint’ model at baseline, as no longitudinal data is available. a. The mean difference in bDMARD dose divided by the mean number of flares compared with the DRESS DGDO arm. The number therefore represents the increase in bDMARD dose that was needed to prevent a flare for this specific tapering strategy. b. The mean difference in the number of flares, divided by the mean difference in bDMARD dose, compared to routine care. The ratio thus represents the number of extra flares that occurred for each extra full dose of bDMARD that is tapered compred to routine care over 18 monhts using this specific tapering strategy. bDMARD: biological disease-modifying antirheumatic drug, DGDO: disease activity guided dose optimisation. [file 13075_2022_2751_MOESM6_ESM.docx]

|  | With baseline predictions cut-off 35% | Without baseline predictions cut-off 35% |
| --- | --- | --- |
| Mean no. of flares | 0.75 (0.55-0.95) | 0.76 (0.56–0.97) |
| Decrease in flares compared to DRESS DGDO | 0.45 (0.16-0.74) | 0.45 (0.15–0.74) |
| Mean bDMARD dose | 0.64 (0.61-0.68) | 0.64 (0.60–0.68) |
| Increase in bDMARD dose compared to DRESS DGDO | 0.10 (0.05–0.16) | 0.10 (0.05-0.16) |
| Percentage of patients flaring | 45% (36%-54%) | 46% (37%-56%) |
| Increase in bDMARD dose per flare prevented vs. DRESS DGDO^a^ | 0.23 (0.15-0.32) | 0.23 (0.15–0.31) |
| Number of extra flares per full bDMARD dose saved vs. routine care^b^ | 1.0 (0.3–1.8) | 1.0 (0.3–1.8) |

***Supplementary Table S3: Simulation results with and without baseline predictions****95% confidence intervals are presented between brackets. The results from external validation in the DRESS trial*(9) *without baseline predictions, for the optimal cut-offpoint of 35% as determined in simulation (see Supplementary Table S2). The rationale for leaving out baseline predictions is that the prediction model cannot truly function as a ‘joint’ model at baseline, as no longitudinal data is available. a. The mean difference in bDMARD dose divided by the mean number of flares compared with the DRESS DGDO arm. The number therefore represents the increase in bDMARD dose that was needed to prevent a flare for this specific tapering strategy. b. The mean difference in the number of flares, divided by the mean difference in bDMARD dose, compared to routine care. The ratio thus represents the number of extra flares that occurred for each extra full dose of bDMARD that is tapered compred to routine care over 18 monhts using this specific tapering strategy. bDMARD: biological disease modifying antirheumatic drug, DGDO: disease activity guided dose optimisation.*
